# Circularity in Europe strengthens the sustainability of the global food system

**DOI:** 10.1038/s43016-023-00734-9

**Published:** 2023-04-17

**Authors:** H. H. E. van Zanten, W. Simon, B. van Selm, J. Wacker, T. I. Maindl, A. Frehner, R. Hijbeek, M. K. van Ittersum, M. Herrero

**Affiliations:** 1 Farming Systems Ecology Group, Wageningen University & Research, Wageningen, the Netherlands; 2 Department of Global Development, College of Agriculture and Life Sciences, and Cornell Atkinson Center for Sustainability, Cornell University, Ithaca, NY USA; 3 Plant Production Systems Group, Wageningen University & Research, Wageningen, the Netherlands; 4 Animal Production Systems Group, Wageningen University & Research, Wageningen, the Netherlands; 5 SDB Science-driven Business Ltd, Larnaca, Cyprus; 6 Department of Food System Sciences, Research Institute of Organic Agriculture FiBL, Frick, Switzerland

**Keywords:** Environmental sciences, Biological sciences

## Abstract

Redesigning the European food system on the basis of circularity principles could bring environmental benefits for Europe and the world. Here we deploy a biophysical optimization model to explore the effects of adopting three circularity scenarios in the European Union (EU)27 + UK. We calculate a potential reduction of 71% in agricultural land use and 29% per capita in agricultural greenhouse gas emissions, while producing enough healthy food within a self-sufficient European food system. Under global food shortages, savings in agricultural land could be used to feed an additional 767 million people outside the EU (+149%), while reducing per capita greenhouse gas emissions by 38% but increasing overall emissions by 55% due to the increased population served. Transitioning the EU’s food system towards circularity implies sequential changes among all its components and has great potential to safeguard human and planetary health.

## Abstract (web summary)

Ensuring that waste or by-products from one process form the input of another is key for food systems sustainability. This study assesses the biophysical potential of redesigning the European (EU27 + UK) food system on the basis of circularity principles. Changes in food consumption, crop production, animal production and fertilizer patterns are considered through scenario analysis.

## Main

The global food system is under multiple pressures. The coronavirus disease 2019 pandemic, political conflicts, depletion of natural resources, biodiversity loss and the threat and consequences of climate change challenge food system sustainability, while the global population continues to grow. In other words, humanity faces a massive paradox: we need food to live, but our way of producing food jeopardizes our potential to produce food. In this article, we explore the question ‘how to supply healthy diets to all people while at the same time safeguarding the planet’s health?’. The answer to this question is complex as today’s food system issues are embedded in a highly complex, dynamic and inter-related system with no ‘silver bullet’ solution^1,2^. A transformation of the food system—covering production, processing, distribution, retailing and consumption—is therefore needed to respect human and planetary health^3^.

One potential game-changing future redesign, which is gaining increased attention, is a circular food system. Redesigning today’s food system towards circularity is also a key strategy of the European Union (EU)^4^. In circular food systems, waste—for example, food waste, human excreta and overconsumption of nutrients—is minimized and, if unavoidable, utilized (recycled) in the most sustainable way^5,6^. For example, during food processing, by-products are produced. If these by-products are used as a fertilizer, the use of artificial fertilizer will potentially decrease, while if by-products are used as feed for farm animals, inedible biomass for humans will be transformed into valuable food, manure and ecosystem services. While several studies assessed the potential of animals as recyclers—showing great environmental benefits^7–10^—a holistic circular food system assessment is missing. Getting a holistic picture is crucial because of the inter-relatedness in the food system, for example, a transition towards more healthy diets in which whole grains are consumed instead of refined grains directly impacts the role of animals as recyclers since less by-products will be available^8^.

**Fig. 1:**
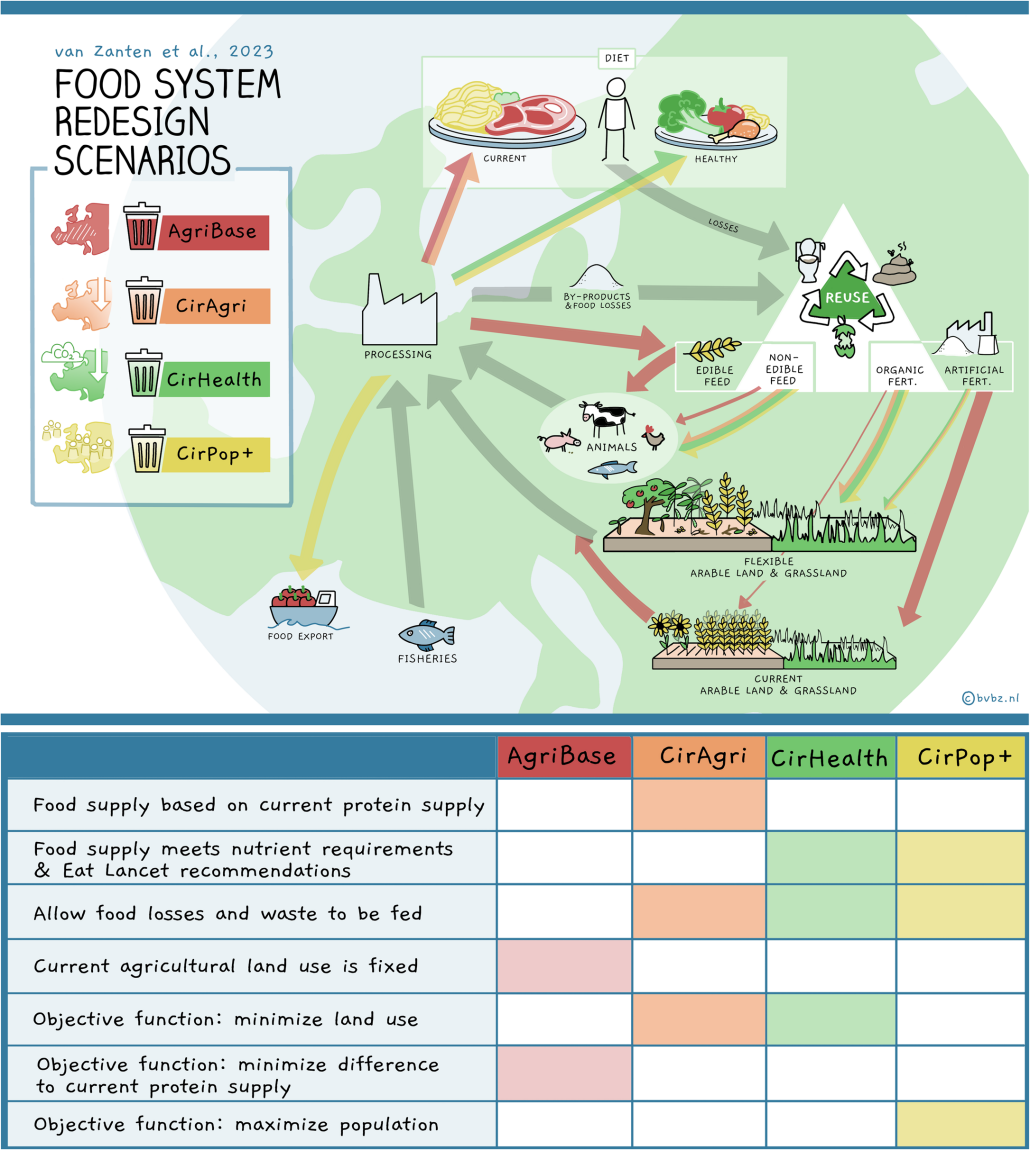
Scenario configuration and illustration. The colours of the arrows and icons on the top left represent the different scenarios in the table. The arrows represent uses of biomass that differ among scenarios. In the case of the grey arrows, the assumptions related to the use of biomass are the same for all scenarios.

In this study we assessed the potential of redesigning the European (EU27 + UK) food system on the basis of circularity principles to secure food availability while minimizing the environmental impact. We designed and assessed three exploratory EU27 + UK food system redesign scenarios varying in their degree of circularity, ranging from changes in supply to more radical transitions towards circular food systems (Fig. 1). Our results provide insights into how the EU27 + UK’s food system can be redesigned in terms of consumption, crop production, animal production and fertilizer patterns, supported by circularity principles.

## Results

### Scenarios

Modelling studies that call for new paradigm shifts for the food system are often based on and locked in our current linear food system, and thus only provide incremental improvements from the status quo^11^. We applied the Circular Food System (CiFoS) model (builds on the work of refs. ^8,12,13^) to assess circularity principles and provide extended alternatives to redesign our current food system to higher sustainability levels. CiFoS is an interactive biophysical data-driven optimization model that facilitates the selection of food system redesigns by minimizing agricultural land use and accounting for greenhouse gas (GHG) emissions while meeting dietary choices.

An agricultural production baseline (AgriBase) and three alternative scenarios were built on the basis of circularity principles (for a detailed configuration, see Fig. 1). The first scenario—the circular self-sufficient agricultural Europe (CirAgri) scenario—solely applies supply-side circularity principles while the other two scenarios—circular self-sufficient healthy diet (CirHealth) and CirHealth Population Plus (CirPop+)— combine both supply and consumption-oriented circularity principles. Consumption, in the CirAgri scenario, meets our current protein supply. Today’s protein supply, on the basis of the statistics of the Food and Agriculture Organization of the United Nations (FAOSTAT), is met on a food group level (grains, tubers, vegetables, fruit, dairy, red meat, poultry, eggs, fish, legumes, nuts/seeds, oil fat, sugar and other). CirHealth and CirPop+ transition consumption to healthier dietary patterns based on, for example, the recommended intake levels per food group derived from the EAT–Lancet guidelines^14^. In addition, CirPop+ aims to use European agricultural land in a way that maximizes the number of people nourished with a circular diet, thereby contributing to global food availability. CirAgri and CirHealth solely focus on the European population and aim to meet their dietary constraints while minimizing land use, to free up land for nature conservation leading, in general, to more biodiversity. All scenarios focus on a hypothetical, self-sufficient European food system and account for GHG emissions.

The production values of the three circular scenarios are compared with AgriBase, a baseline production scenario that matches empirical data^15^ related to, for example, current crop production systems for domestic use and export with associated areas, while minimizing the difference with the current food supply^16^ (objective function).

### Sustainability potential of circular European food systems

**Fig. 2:**
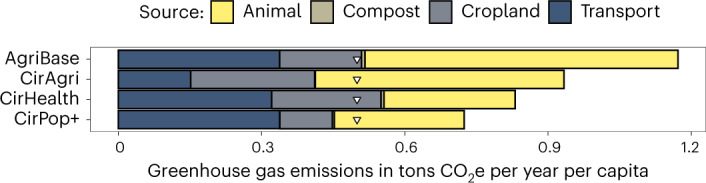
Source-specific GHG emissions under different degrees of circularity. The AgriBase scenario shows the highest emission levels, followed by CirAgri, CirHealth and CirPop+. Triangles indicate the safe operating space of the planetary boundaries’ framework food production per capita per year (511 kg CO_2_e; ref. ^14^).

**Fig. 3:**
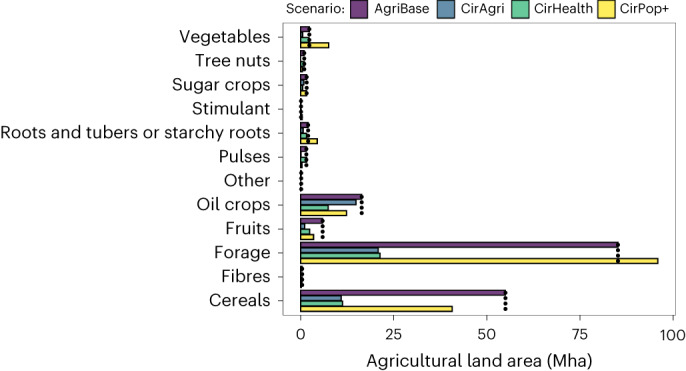
Agricultural land use per crop group for the different scenarios. Dots represent current land use. Crops are used for food and feed purposes. Fodder crops include clovers, alfalfa, legumes and grass.

**Extended Data Fig. 1:**
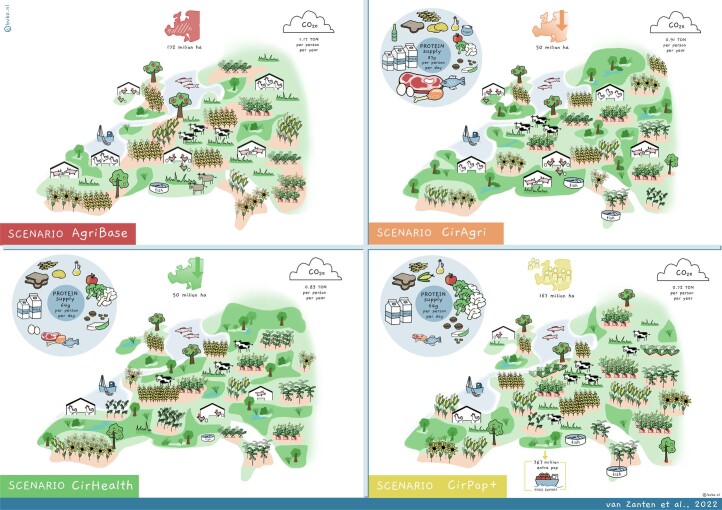
Visual abstract. This figure provides a visual abstract of the paper.

The AgriBase results show an annual total of 644 million tons of CO_2_ equivalent (MtCO_2_e) (1.17 tons CO_2_e per person per year based on 2020 population numbers) with a use of 172 Mha of agricultural land (Figs. 2 and 3 and Extended Data Fig. 1). The agricultural land area, crops cultivated and corresponding yields match FAOSTAT data and therefore provide a ‘validated’ baseline (Fig. 3). In comparison, CirAgri reduces GHG emissions by 22% and agricultural land use by 71% while meeting current protein supply. In CirAgri, annual GHG emissions were 515 MtCO_2_e (0.91 tons CO_2_e per person per year) with an agricultural land use of 50 Mha. CirHealth, with changes in supply side and consumption, reduced GHG emissions by 29% (annual total 458 Mt; 0.83 tons CO_2_e per person per year) and agricultural land use by 71% (50 Mha). These figures show that redesigning the EU27 + UK food system allows the EU to be self-sufficient (no import and export), while reducing GHG emissions and agricultural land use. People outside the EU currently depend, to some degree, on European agricultural exports, and the CirPop+ scenario shows that the EU27 + UK can provide a healthy diet to another 767 million people (+149% compared with the EU27 + UK population). Even here, GHG emissions are reduced by 38% per capita (0.72 tons CO_2_e per person per year) but overall emissions increased by 55% (annual total of 998 MtCO_2_e). Total agricultural land use differed 3% from the current situation (to 167 Mha). Therefore, the combination of avoiding overconsumption and healthy eating while recycling residual food system streams has the potential to greatly improve human and planetary health.

### Agricultural land use changes needed for circularity

Of Europe’s 172 Mha of agricultural land, 105 Mha are croplands used for food and feed and the other 67 Mha are grasslands. In the circular scenarios, the area of cropland used was reduced by 53% for CirAgri (50 Mha), 53% for CirHealth (49 Mha) and by only 3% for CirPop+ (102 Mha). The area of grassland was reduced with almost 100% for CirAgri and CirHealth and by 2% for CirPop+ (66 Mha).

#### Cropping systems

European crops require crop rotations, resulting in a combination of crops grown over the years. Figure 3 shows the selected crops within the crop rotations, illustrating that the EU27 + UK’s current cropland is dominated by the production of cereals (for food and feed) and oil crops (AgriBase; Fig. 3). About half of arable land is used to produce cereals (mainly wheat and barley) and about one-fifth to produce oil crops (mainly rapeseed and sunflower) for food and feed purposes. In general, CirAgri and CirHealth show a trend towards less cereals and less fodder crops. Furthermore, they show increased diversity—mainly in the CirHealth scenario—with relatively more land used for pulses (soybean, chickpea, beans and lentils), sorghum, green and red vegetables, and tropical fruits.

#### Fertilizer use

**Fig. 4:**
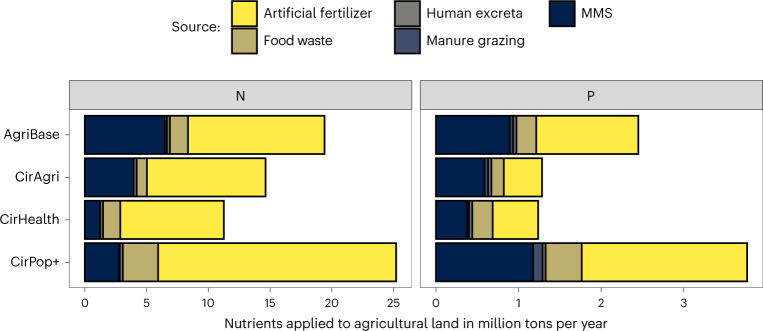
Amounts of N and P applied to agricultural land, classified per source. Compared with AgriBase, the use of N and P is reduced in CirAgri and CirHealth. In CirPop+, the total use of artificial fertilizer (both N and P) increases as insufficient organic fertilizers are available to fertilize the crops needed to nourish the increased population.

In total, AgriBase estimates 12.3 Mt artificial fertilizer application (nitrogen (N) + phosphorus (P) applied)^17^, supplemented with manure, compost and currently used human excreta. N and P use are both reduced in CirAgri and CirHealth (Fig. 4). In CirPop+, the total use of artificial fertilizer (both N and P) is relatively high compared with the other two circular scenarios as insufficient organic fertilizer is available to fertilize the crops needed to nourish the increased population. When animal numbers are decreased (CirHealth and CirPop+), the use of food waste as organic fertilizer over feed for animals is prioritized (see also Supplementary Data ‘Biomass flows’), while otherwise, food waste is largely used as feed and the manure is used as fertilizer (CirAgri).

#### Livestock systems

**Fig. 5:**
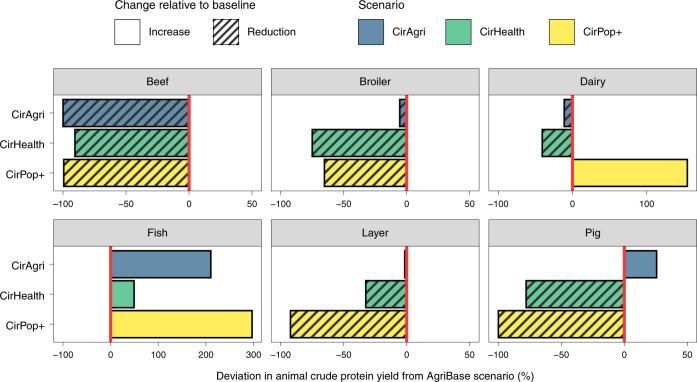
Total animal crude protein yield per year per scenario relative to the AgriBase scenario (percentage). The AgriBase scenario reflects the animal crude protein yield needed to meet current protein supply. CirAgri meets the current protein supply per food group, resulting in relatively small changes in crude protein yields, while CirHealth and CirPop+ (representing healthy diets) result in large reductions in animal crude protein yields.

Applying circularity principles, that is, feeding animals with by-products, food waste and grass, necessitates a radical redesign of the livestock sector (Fig. 5). In the CirAgri scenario, which meets the current animal protein supply, animal protein yields are relatively stable, with only fish and pig production clearly increasing while beef production decreases. CirHealth and CirPop+ show large reductions in beef cattle (91% and 99%, respectively), pigs (78% and 100%, respectively), broilers (79% and 73%, respectively) and layers (33% and 93%, respectively). Dairy and fish show relatively small changes in the CirHealth scenario, while they largely increase in the CirPop+ scenario.

Feed–food competition is reduced in all circular scenarios (Supplementary Fig. 9). Feed consists of food losses and waste, grass, by-products and fodder crops. The share of food losses and waste in animal feeds largely differs across animal types: from zero in ruminant feed to large shares in fish, chicken and pig feeds (Supplementary Fig. 9 and Supplementary Data ‘Animal feed’). Although feed-food competition decreases, animals are still largely fed with fodder crops in all circular scenarios, especially in CirAgri. Without fodder crops there is insufficient non-food-competing feedstuff available to feed the number of animals needed to meet current supply (in line with refs. ^18,19^) of animal protein (CirAgri) or to meet all nutritional requirements (for example, vitamin B12) (CirHealth and CirPop+).

### Consumption changes needed for circularity

Food production from AgriBase plus imports reflect current FAOSTAT protein supply levels in the EU27 + UK, but not fully as production and consumption data of FAOSTAT are not harmonized^20^. When subtracting losses from supply to obtain the actual intake, protein consumption is 69 g of protein per person per day.

**Fig. 6:**
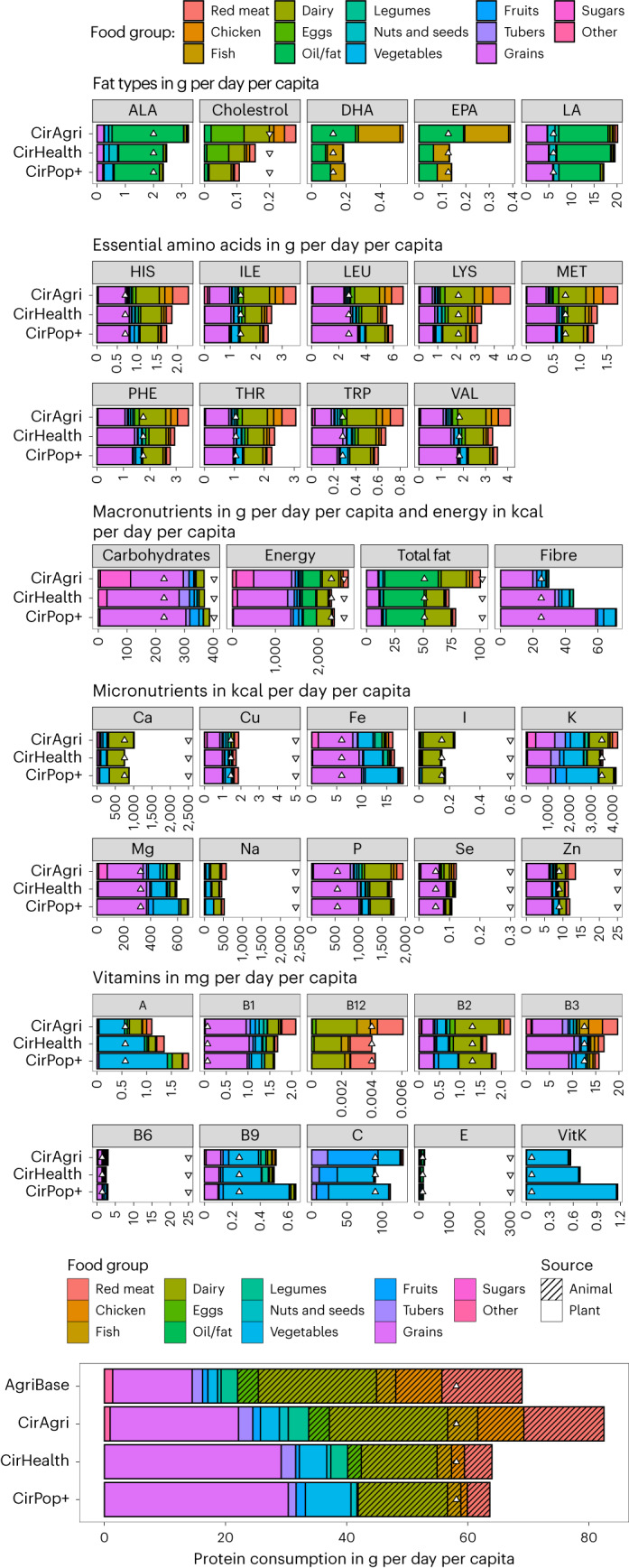
CirAgri represents the current supply while CirHealth and CirPop+ represent a healthy diet. Triangles show minimum (upwards) and maximum (downwards) requirements per capita per day based on the EFSA. Results for the AgriBase for protein requirements indicate to what extent EU27 + UK production plus imports matches the FAOSTAT protein food supply.

In the CirAgri scenario, sufficient food can be produced to meet current protein supply (103 g per person per day^15^) resulting in a protein intake of 83 g of protein per person per day in a circular European food system without the need for importing food. In the CirHealth and CirPop+ scenarios, food intake meets nutrient requirements and the recommended food intake levels of the EAT–Lancet guidelines, resulting in 64 g of protein per person per day (Fig. 6).

In the CirAgri scenario, the majority of macro- and micronutrients values fall between the minimum and maximum range of recommended intake. Only energy and cholesterol exceed the maximum recommended intake (Fig. 6). Compared with the recommended food intake levels by the EAT–Lancet guidelines that account for foods instead of nutrients (essential to ensure that foods that limit the risk for dietary-related diseases are consumed) current consumption patterns show an excessive consumption of sugar, red meat and milk, with eggs at the upper limit, while vegetable consumption is too low (Supplementary Fig. 11). Our results, therefore, support the literature on unhealthy dietary patterns^21^.

In CirHealth and CirPop+, all macro- and micronutrients supplied fall within the bounds of recommended intakes, thus providing a basis for nutritionally adequate diets (Fig. 6). In none of the scenarios is protein the limiting factor, while energy is, for example, limiting, meaning that energy is limited when we minimize land use (CirHealth) or optimize the number of people to feed (CirPop+). In general, the results show that the limiting of macro- and micronutrients largely depends on the consumption of animal source foods (ASFs) in our dietary scenarios. A transition towards a healthier diet requires a change in the ratio between animal proteins and plant proteins: from 60:40 in the current situation (CirAgri) to 37:63 in CirHealth and 34:66 in CirPop+. The overall reduction in animal protein intake per capita is 51% in CirHealth and 55% in CirPop+, that is, from 49 g per person per day to 24 and 22 g per person per day, respectively. While most animal proteins were reduced, the majority was due to reduced red meat and chicken meat consumption.

## Discussion

This study shows that redesigning the food system on the basis of circularity principles has great potential to feed a population with sufficient, healthy food, while reducing the environmental impact. Agricultural land use was largely reduced, just as CO_2_ emissions, albeit that the latter were still above the boundary of 0.5 tons CO_2_e per person per year recommended by ref. ^14^. Additionally, we showed that a transition towards circular food systems could improve food provisioning to other parts in the world. Our results revealed that the following elements are key for an EU27 + UK food systems transition.

### Current crop rotations limit the circularity potential in the EU27 + UK food system

Crop rotations are essential from an agro-ecological perspective, for instance to maintain soil quality and manage soil-borne pests and diseases^22,23^. In our model simulations, therefore, some food crops were assumed to be grown, for example, with a maximum of once every 6 years (for example, sunflower) or every 3 years (for example, potatoes); for the other years, other crops must be selected. Certain food crops needed to fulfill human nutritional requirements steer land use—showing the need for food crop diversification. We noted in our study that crop rotation acted as a driver of production of fodder crops, such as alfalfa and clover, as without fodder production, land would have been left uncovered in the remaining years of a crop rotation. This is because the potential rotation combinations to produce more foods for direct human consumption in the CiFoS model were limited by the range of food crops included (and currently cultivated on a relatively large scale).

### Animals play an important role in EU27 + UK circular food systems, although their numbers are largely decreased

This is in line with studies that assessed the role of animals as recyclers, ranging from 16 to 40 g of protein per person per day^8,13^. Among others, total energy intake, eicosapentaenoic acid (EPA), iodine, vitamin B12 and calcium are limiting in CirHealth and/or CirPop+. Notably, vitamin B12 is only produced from animal proteins and therefore drives consumption and production. Vitamin B12 intake recommendations, however, vary largely, that is, from 2.4 µg (World health Organization) to 4 µg (European Food Safety Agency (EFSA)) per adult per day^16^. Using alternative nutrient requirements or supplements could impact the ratio between animal proteins and plant proteins.

### Organic and mineral fertilizers needed in circular EU27 + UK food systems

We showed that the amount of organic fertilizers available is not sufficient in circular EU27 + UK food systems to fertilize the crops needed to nourish the population. Especially when livestock numbers go down drastically, much less organic manure for fertilization is available, increasing the need for alternative organic fertilizers such as green manure or human excreta. On the basis of current use, we assumed that only 36% of all human excreta was available as fertilizer^17^. Nutrient recovery from human excreta is highly dependent on sanitation systems to avoid potential health hazards from pathogens, organic pollutants and heavy metals^24,25^. Allowing for redesign of sanitation systems in the CiFoS model, therefore, might result in a large pool of unused nutrients, in particular of P.

### Reductions in grassland areas are likely in circular food systems and could potentially enhance biodiversity

The use of grasslands is reduced in the circular scenarios. The need for grasslands increases only in case of global human nutritional shortages (CirPop+), in line with earlier findings^26^. Reductions in grasslands offer the opportunity for rewilding to enhance biodiversity. Another alternative would be to partly maintain grassland but with low livestock densities, with the aim of contributing to multiple ecosystem services such as water cycling, carbon storage and sequestration next to biodiversity conservation^27^.

### Consumption patterns in EU27 + UK towards healthy diets

Consumption patterns ought to shift towards healthier diets, including increased amounts of fruits and vegetables, wholegrain products, legumes and plant-based oil sources. While our scenarios CirHealth and CirPop+ provide all the required components for healthy diets, healthy eating goes beyond this. For example, foods need to be combined effectively to enhance bioavailability^28^, and appropriate processing methods must be chosen that preserve and make important nutrients available^29,30^. To facilitate this, a diversity of measures is required, from appropriate processing to changes in food environments, as well as changes in (relative) pricing, accompanied by education and information activities^31^.

### Potential impact of self-sufficiency and circularity on indirect land used change (LUC) emissions

LUC emissions related to imported food and feed account for 30% of GHG emissions of European diets^20^. A circular, self-sufficient food system avoids import by default and therefore transitioning the European food system will most likely reduce indirect LUC emissions. In this study, we considered only direct effects related to the European food system. Potential indirect effects, for example, land savings outside the EU27 + UK when soybeans are no longer imported, are highly uncertain as data (for example, FAOSTAT inconsistencies) and methodologies (for example, allocation of co-products) are not harmonized^20^ and were therefore not considered in this study.

### Model limitations and data availability

Our study focused on food system interventions related to circularity principles. Notably, circularity must be applied beyond the food system and include the feed–food–fibre–fuel competition. Furthermore, it should be noted that many interventions offering potential to enhance sustainability of the food system exist, for instance, increasing the use of precision management, and changes in feeding and breeding strategies impacting animal productivity. The model moreover includes many data uncertainties due to limited availability and inconsistency of data and data sources: for example, availability and usability of food losses and waste, statistical data on fertilizer use in relation to crop productivity levels, harmonization of agricultural land area maps for different years and harmonization between food-based dietary guidelines among the EU27 + UK. We therefore stress the need for data recording, collection and harmonization, not only for the EU27 + UK but on a global scale.

### Long-term systemic planning is required

Humanity is facing the combined urgent challenges of making healthy diets accessible to all and safeguarding the planet’s health. Previous studies have already shown the great potential of waste reductions and/or transitions towards more healthy diets^3^_._ The study of ref. ^3^ showed that a diet according to World Health Organization guidelines reduces GHG emissions by 29% and cropland use by 7%. The study of ref. ^32^ showed that replacing 25–50% of animal-derived foods with plant-based foods in the EU could reduce GHG emissions by 25–40% and the use of cropland by 23%. We show that redesigning the EU27 + UK food system on the basis of circularity principles can even further reduce agricultural land use and GHG emissions, while simultaneously enhancing food availability. Transitioning the food system towards circularity implies sequential, systemic changes among all of its components, jointly shaping a radical redesign of the food system. Circular food system redesigns do not depend on future technologies—they can largely be implemented tomorrow—but they do depend on social acceptance and a radical transformation of the economic sector^33^. To achieve the needed changes, stakeholders need to be aware of the urgency and reach consensus on the direction of redesigns. Food systems research can play a crucial role by exploring redesigns and assessing the impacts and trade-offs of alternatives. Our explorations of radical redesigns of current European food systems provide dots on the horizon to discuss and eventually agree upon options to realize long-term, sustainable human and planetary health^34^.

## Methods

Building on the models of refs. ^8,12,13^ and the concepts of refs. ^9,10,26,35–40^ we developed the CiFoS model to grasp the full complexity of food systems. Circularity is a systemic solution: waste streams—for example, food waste, human excreta and overconsumption of nutrients—are reduced and, if unavoidable, utilized in the most sustainable way. Furthermore, by-products are produced during food processing (for example, wheat middlings when producing flour). Using these by-products and waste streams as compost can reduce the need for artificial fertilizers, while if by-products and waste streams are used as feed for farm animals, inedible biomass for humans could be transformed into livestock products and manure. In both cases, ecosystem services should increase due to improved soil fertility and less pressure on land^5,27,41^. Avoiding and reusing waste and by-product streams to close biomass and nutrient cycles are the key principles of circular food systems.

**Extended Data Fig. 2:**
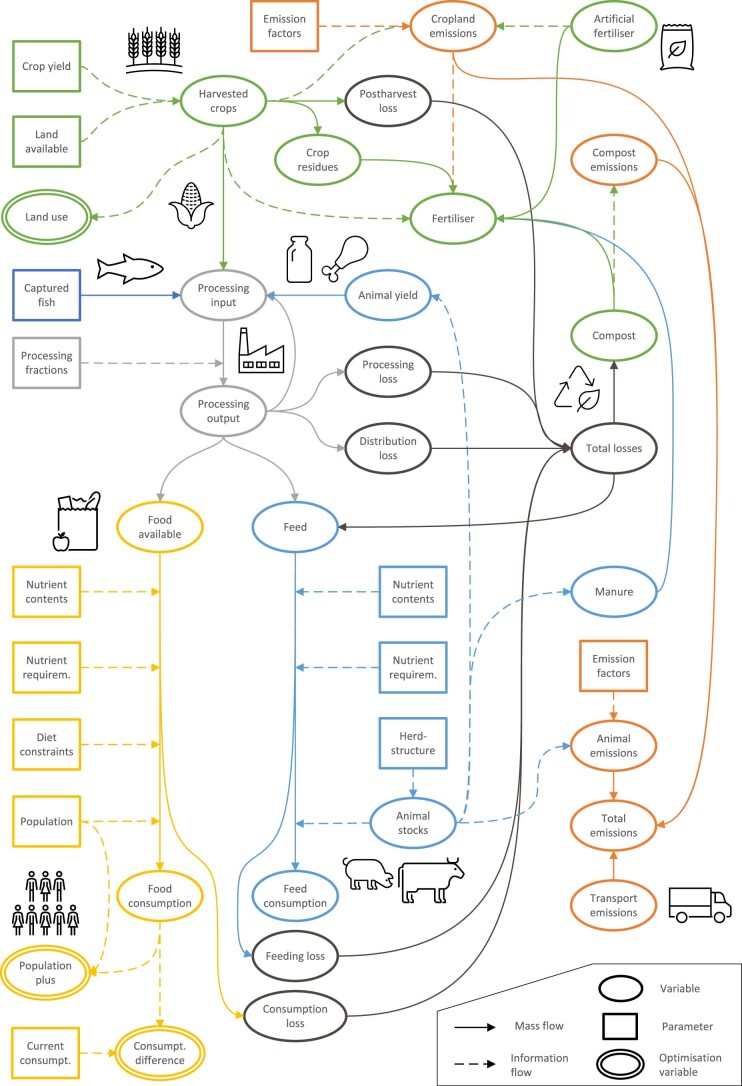
Visualization of the Circular food systems (CiFoS) model. The figure illustrates the different modules within the model - human-system, crop-system, farmed-animal-system, fisheries, residual streams, transportation, and GHG emissions.

The CiFoS model was developed with a generic set-up, making it applicable to different regions. We focused on the EU27 + UK and used European-level data to run the model. CiFoS is a biophysical data-driven food system optimization model coded in General Algebraic Modeling System that embraces circularity principles due to its unique model structure (Extended Data Fig. 2). The CiFoS model accounts for the potential to use natural processes and cycles to ensure that waste or by-products from one process form the input of another process. For example, human inedible by-products can be used as a fertilizer or as feed for farmbanimals. CiFoS facilitates the choice of food system redesign by minimizing a selected environmental impact (for example, land use or GHG emissions) or maximizing the population fed, as objective functions for meeting human dietary needs, using constraints on nutrients, protein and energy intake per capita. The model therefore selects a combination of food items from plants, farm animals or captured fish that minimizes land use. The CiFoS model contains various scales: from the EU27 + UK, or a country to an agro-ecological zone. The CiFoS model components consist of several modules—human system, crop system, farmed animal system, fisheries, residual streams, transportation and GHG emissions. These are described in more detail below.

### Human nutrition systems

The model considers three dietary constraints in different scenarios—the current food supply, nutritional requirements and food group requirements—with the aim of distinguishing between the current diet and a healthy diet. Current food supply is based on the FAOSTAT, averaged between 2010 and 2019. In the CirAgri scenarios, the current total protein supply per country is met per food group (grains, tubers, vegetables, fruit, dairy, red meat, chicken, eggs, fish, legumes, nuts/seeds, oil fat, sugar and other). For nutrient requirements, the CiFoS model accounts for the 42 most important macro- and micronutrients (Table 1). In the CirHealth and CirPop+ scenarios, the daily recommended nutrient requirements advised by the EFSA for the EU27 + UK are met to ensure a nutritious diet. The nutritional content of each food item was based on the United States Department of Agriculture (USDA) National Nutrient Database for Standard Reference, Legacy (2018). Nutritional values for all plant-based food, milk and eggs are directly available on the USDA database. For meat products^12^, we calculated the average nutrient content per kilogram carcass weight based on relative cut weights.

**Table 1:** All 42 nutritional values, concerning human nutrition, accounted for by the CiFoS model

General	Macronutrients	Fat types	Micronutrients	Vitamins	Essential amino acids
Fresh weight Energy	Protein Fat total Carbohydrates Fibre	Linoleic acid (LA) Alpha-linolenic acid (ALA) Docosahexaenoic acid (DHA) Eicosapentaenoic acid (EPA) Cholesterol	Natrium (Na) Potassium (K) Calcium (Ca) Phosphorus (P) Magnesium (Mg) Iron (Fe) Copper (Cu) Selenium (Se) Zink (Zn) Iodine (I)	A B1 B2 B3 B6 B9 B12 C D E K	Histidine (HIS) Isoleucine (ILE) Leucine (LEU) Lysine (LYS) Methionine (MET) Phenylalanine (PHE) Threonine (THR) Tryptophan (TRP) Valine (VAL)

In addition to nutrient requirements, food intake constraints per product and/or food family were included in CirHealth and CirPop+ based on the reference range of the EAT–Lancet dietary guidelines^14^ to ensure that the healthy circular diet remains diverse and respects health advice related to maximum intake of, for example, red meat, or minimum intake of, for example, fibre (Table 2).

**Table 2:** Minimum and maximum intake of foods or food groups based on the EAT–Lancet dietary guidelines. All mass units are in fresh weights

	Minimum	Maximum	Unit
Grains	0	60	% of energy uptake
Tubers	0	100	g per day per capita
Vegetables	200	600	g per day per capita
Fruit	100	300	g per day per capita
Dairy	0	500	g per day per capita
Red meat	0	28	g per day per capita
Chicken	0	58	g per day per capita
Eggs	0	25	g per day per capita
Fish	0	100	g per day per capita
Legumes	0	225	g per day per capita
Nuts/seeds	0	75	g per day per capita
Sugar	0	31	g per day per capita

### Cropping system including land use

The AgriBase scenario represents the current (reference year 2010) crop production systems and associated crop yields, areas and fertilizer use in Europe. In the other scenarios, the type and land area of crops selected by the model depend on their potential to be grown somewhere (that is, the climate soil zones), the current crop yields in the climate and the soil zone, crop rotation requirements, fertilizer requirements and the nutrients they provide for the human diet.

#### Land availability

The total area of agricultural land available in the EU27 + UK is 171.7 Mha (FAOSTAT). Agricultural land is split into three distinct land use classes: cropland (including temporary grasslands), permanent pasture and rangelands. Pasture and rangelands are both permanent grasslands, where the nutritional content (for example, protein and energy) of the harvested biomass is assumed to be the same for both grassland types (as systematic data on differences are lacking). The land cover map for cropland was taken from ref. ^42^ and represents the year 2005. The grassland cover types ‘grazing’, ‘pasture’ and ‘rangeland’ were chosen from HYDE for the reference year of 2010 (ref. ^43^). In a harmonization process, we combined the different layers to match the desired four land use types: cropland, temporary grassland (‘pasture’ or rangeland that overlapped with cropland), permanent pasture (‘pasture’ that does not overlap with cropland) and rangelands (‘rangeland’ that does not overlap with cropland).

For the AgriBase scenario, the crop areas are scaled to match the current area per crop and land use type on a country level using FAOSTAT data. In all other scenarios, the model selects the area and type of crops cultivated. Climate soil zones determine which crops can be grown where. An exception was made for rice due to its dependency on groundwater levels, which could not exceed its current acreage. Furthermore, crops that currently occupy less than 50 ha in a climate soil zone or less than 5,000 ha of the total EU27 + UK arable land area cannot exceed its current acreage. All our crop-related data were spatially extracted on the basis of country climate soil zones. These zones were based on: country borders of EU27 + UK (FAO GAUL: Global Administrative Unit Layers 2015, Administrative level 0), agro-ecological zones (GAEZ version 4, 33 classes)^44^ and soil zones based on ‘Intergovernmental Panel on Climate Change (IPCC) default soil classes derived from the Harmonized World Soil Database’, which we aggregated to clay, organic, sand, other soils, wetlands and other^45^. By overlaying these three spatial layers, we created model-specific zones that were the base unit for all crop- and grassland-related data extraction processes. With the zones- we aimed to represent geographic areas with homogeneous biophysical characteristics and natural resources such as climate (that is, temperate, tropic and boreal), moisture regime (wet and dry) and soils (soil type and soil organic carbon), which strongly drive agricultural productivity and natural processes such as leaching. The choice of crop depends on whether there is production data in a specific zone. If there is no yield, the crop is considered unsuitable and cannot be selected by the model. Constructing these zones allowed us to have a more accurate representation of the potential crop and grassland extension. In total, the EU CiFoS model accounts for 850 climate soil zones.

#### Crop choice

CiFoS includes 43 food crops and eight fodder crops, including three different grass types (temporary, permanent and rangeland). Crops that can be used either as a feed or a food source (for example, maize) fall, depending on their use, under the group forage or cereals. Yields and areas per crops were based on the Global Spatially Disaggregated Crop Production Statistics Data for 2010 (version 2.0), referred to as SPAM^46^. The current yields are represented by SPAM, which uses a 3 year average of yields and harvested areas from 2009 to 2011. SPAM is based on statistical crop data from a country down to a municipality level. Tree nuts and three vegetable types (leafy green, red/orange and other vegetables) were disaggregated from the SPAM crops data using the EARTHSTAT dataset ‘Harvested Area and Yield for 175 Crops’^47^. The disaggregation of these crops was needed as SPAM pooled tree nuts together with spices in the crop ‘other’ while all vegetables were summarized in one crop ‘vegetables’. To do this, we first defined the final crops: the initial crop ‘rest’ became ‘rest’ + ‘treenuts’ and crop ‘vegetables’ became ‘green’, ‘red’ and ‘other vegetables’. Then, all the disaggregated crops of SPAM were classified to the nested crops of the EARTHSTAT dataset (tree nuts would be matched to hazelnut, walnut and so on). In the last step, the ratio between these disaggregated new crops was determined per pixel to derive a factor, which was used to disaggregate the crop parameter (harvested area, production and yield) of the aggregated SPAM crop. As a result, we incorporated crop data from tree nuts, red, green and other vegetables. Fodder crops not represented by SPAM were additionally added from the more complete but older EARTHSTAT data. The EARTHSTAT contains the complete list of FAO crops but only represents the year 2000 based on the era 1997–2003. Therefore, we only used the EARTHSTAT dataset when SPAM was lacking disaggregated crops that seemed nutritionally or agriculturally highly relevant (vegetables, tree nuts and several forage crops). All crops were assigned to the crop groups defined in SPAM, which were then aligned with the food groups from the EAT–Lancet dietary guidelines. The SPAM data were used for four reasons: first, they cover sufficient crop types to develop a food system model at the European level. Second, SPAM provides a sophisticated spatial crop allocation method^42^. Third, SPAM data provide the most recent crop distribution data, and fourth, the data incorporate a sufficient level of detail regarding technology levels (irrigated and rainfed-high/low/subsistence^46^) for use in future studies.

#### Crop rotations

Most crops are grown in rotations to maintain soil health. In the CiFoS model, we account for crop shares by transforming a crop rotation combination of several years in a yearly average area shares per crop (for example, a 1:4 rotation with four crops gives 0.25 ha per year for each crop). The model therefore does not provide a dynamic outcome, but a static average. The frequency of the cultivation of the same crop in time is derived from ref. ^48^. We do not account for crop sequence constraints.

#### Crop fertilization

Crop fertilization includes N and P. Fertilizer requirements were derived by multiplying the harvested part of the crop and the aboveground residues by the P concentration and an unavoidable loss fraction of 12.5% (ref. ^49^). A similar approach was followed for N, but losses (related to aboveground biomass) due to volatilization, leaching and run-off were calculated based on IPCC equations^50^. The ratio between N and P in all organic fertilizers was assumed fixed. In other words, if an organic fertilizer is used, this fertilizer has a certain N to P ratio.

#### Fertilizer types

To select a crop, the model also needed to meet the fertilizer requirements using by-products, manure, compost, human excreta and artificial fertilizer. Crop fertilization in the AgriBase scenarios was based on the current amounts of N+P artificial fertilizer of 12.3 Mt (EuroStat) and 3.2 Mt of sludge (fresh matter, see the ‘Human excreta’ section), while in the CirAgri, CirHealth and CirPop+ scenarios, artificial fertilizer was optional and complementary to organic sources. The N and P content of animal manure is calculated as a function of animal nutrient intake and nutrients retained in meat, milk or eggs. Similarly, the N and P content of compost is calculated as a function of nutrients in food waste (see the ‘Food waste’ section). The N and P contents of crop residues are based on the CVB database^51^. We assumed a long-term equilibrium situation, in which organic fertilizers are used for many years to achieve a steady state of the soil between nutrient inputs and uptake or losses^52^. This implies that we assumed all N and P in organic manures to be available to the crop. Besides nutrient supply, the use of organic residue streams as fertilizer may have other, more difficult to quantify, benefits for soil quality such as improved soil structure, increases in water infiltration and water holding capacity, or improved soil life^53^. Ongoing research on establishing thresholds for soil organic matter content^54^ may provide minimum quantities of organic amendments to be returned to the soil. As such values do not exist yet, we assumed all crop residues stay on the field.

### Farmed animal systems

The animal system includes livestock (dairy, beef, pigs, broilers and layers) and farmed fish (Atlantic salmon and Nile tilapia) on the basis of ref. ^12^. The two fish systems are a proxy for freshwater and saltwater fishes. Livestock systems include three productivity levels (high, medium and low), while farmed fish only reflect the current average production level. For each animal, a fixed relation between animal productivity and nutrient requirement (for example, protein and energy) is assumed. The nutritional requirements of livestock and farmed fish can be found in ref. ^12^ and Supplementary Information. The model included the parent stocks (for example, sow in a pig system) and reproduction stocks (for example, heifer in a dairy system) to account for the animal’s entire lifecycle. Thus, a fatting pig can only be produced when the nutritional requirements of the fatting pig, the parent stock and reproducing stock are fulfilled, while also accounting for the intake capacity of each animal. To meet those requirements, the model can select different feed ingredients, ranging from co-products, food waste, grass resources, animal by-products and high-quality biomass such as grains, which humans can also consume. The nutritional value of each ingredient for livestock was obtained from ref. ^51^ and the nutritional value of each ingredient for farmed fish was obtained from the International Aquaculture Feed Formulation Database^55^. Ruminants were not allowed to feed on any food losses or waste. The final feed ration is a model outcome. The amount of ASF produced by diverse systems is determined by (1) the quantity and quality of biomass and grass resources available for farm animals, (2) the capacity of these animals to convert these biomass streams into ASF and (3) the nutritional value of the animal-based products and demands for the human diet.

### Fisheries

In addition to aquaculture, the model includes capture fisheries. Capture fisheries provide fish for human consumption and fish by-products (for example, fish meal), which could be fed in the animal systems. Quantities of capture fisheries (that is, harvested fish in tons of fresh fish) are based on ref. ^56^. The amount of fish that can be harvested from a waterbody depends on the production capacity of its fish stocks, which in most EU shared waters is impaired due to overexploitation^57,58^. As the production capacity of a waterbody should be sustained or even restored in a sustainable food system, landings were assumed to be limited to the maximum sustainable yield (MSY) implemented in EU legislation. This MSY represents the highest achievable landings without long-term negative impacts on the population, considering both harvested biomass and fish mortality (EU, 2013). A distinction was made between the edible yield fraction of all landed food-grade fish and their non-edible by-products to account for feed–food competition. The MSY landings of 100 stocks of 16 species in the Northeast Atlantic^59^ were quantified to estimate EU MSY landings. Subsequently, the share of these landings available to EU member states on the basis of the current quota distribution was quantified (EU council, not publicly available). The 16 species were most relevant in terms of biomass landed in 2016, for which coherent data on MSY landings and quota distributions was available. In 2016, the 100 stocks provided 75% of total EU landings^60^, the remainder originated mainly from the Mediterranean and Black seas.

### Residual streams

#### Crop residues

In addition to the main crop yield, cereal and oil seed crops also produced a residue crop yield; these are based on data from the USDA PLANTS Database^49^. In our study, crop residues are products that are left on the field after harvesting and are not reused.

#### By-products

Crop and animal products require processing into human food. Quantities of by-products (for example, wheat bran and wheat flour) from crops (for example, wheat) were calculated using technical conversion factors^61^. Technical conversion factors represent the fraction of main product (for example, wheat flour) and by-product (for example, wheat bran) resulting from each process (for example, wheat milling). The availability of animal by-products was a fraction of the predicted live weight output of each livestock system^56^. By-products could be used as animal feed or soil amendments.

#### Food losses and waste

Food waste occurs at all stages along the supply chain, including post-harvest, processing and packaging, distribution and retail, and consumption. Post-harvest, processing and packaging, and distribution waste occur in the country of production. Consumption waste occurs in the country of consumption. The percentage of food lost or wasted varies depending on the stage of the supply chain and type of product^62^. Of the consumption waste, 35% was assumed to be available for animals as a wet feed, which is considered achievable if the feeding of food waste to animals were to be legalized, on the basis of the example in Japan^12^. In the model, food losses and waste could only be consumed by monogastric animals and fish due to food safety risks.

#### Manure

In addition to producing food for meat, milk and eggs, livestock also produce manure, which can be used as a fertilizer for crops and grassland. All manure was assumed captured in a manure management system (MMS) except that of grazing ruminants. Manure captured in a MMS could be exported, to be applied on, for example, arable land within the country of production (see above for explanation of fertilizer value). Grazing ruminants excrete manure directly onto grassland; the proportion of manure excretion onto grassland is a function of grazing selected in the model.

#### Human excreta

According to ref. ^63^, 36% of sewage sludge is currently used in agriculture in the EU27 + UK. The rest is mainly disposed of by incineration or used for land fill or compost. We used this 36% in all our scenarios. The potential to use the nutrients in the sludge as a fertilizer is thus not fully exploited. The nutrient contents for sludge were assumed to be 7.5% and 1.2% of N and P, respectively^64^.

### Transportation

Food, feed and by-products can be transported by truck between EU27 + UK countries, while food waste and grassland must be used in the country of production. It was assumed that crop and livestock products are processed into food, feed and by-products in the country of production and transported to the country of consumption after processing.

### GHG emissions

#### GHG emissions from animal production system

IPCC tier 2 methodologies were followed to calculate GHG emissions. GHG emissions from terrestrial animals (dairy, beef, pig, broiler and layer) included CH_4_ and N_2_O from manure management, as livestock manure is a source of CH_4_ and N_2_O emissions. Methane emissions from manure management were calculated by multiplying volatile solid excretion by the methane conversion factor (that is, the conversion factor for each MMS), B0 (that is, the maximum methane producing capacity for manure) and 0.67 (that is, the conversion of methane from m^3^ to kg CH_4_). Volatile solid excretion was calculated using the digestibility of protein and organic matter of feed consumed by the animal species. The feed consumed by the animal is a model result. N_2_O emissions from manure include direct and indirect emissions, the latter resulting from the volatilization of ammonia and nitrogen (di)oxide. N excretion was calculated by subtracting N retained in meat/milk/eggs from the N intake. To calculate N_2_O emissions, N excretion is multiplied by the respective emission factor, which varies depending on species and housing system. N_2_O emissions from farmed fish included N_2_O emissions from the aquaculture system. The N in unconsumed feed and excreta (N intake minus N retained in body tissue) was multiplied by 1.8% and converted from N to N_2_O (ref. ^50^).

In addition, for ruminant systems, CH_4_ from enteric fermentation and N_2_O from grassland fertilization were considered. Methane emission from enteric fermentation was calculated by multiplying gross energy intake by Ym (that is, the percentage of gross energy in feed converted to CH_4_) and dividing by 55.65 (that is, the gross energy content of methane) (IPCC tier 2 approach). Nitrous oxide emissions from grassland included direct and indirect emissions (the latter resulting from the volatilization of ammonia and nitrogen (di)oxide and the leaching of nitrate) from N fertilization and manure excretion while grazing.

#### GHG emissions from cropping systems

Cultivating crops contributes to N_2_O and CO_2_ emissions. The IPCC tier 1 methodology was followed. N fertilization of crops, in relation to fertilization type, soil type and climate result in different N_2_O emissions. N_2_O emissions include direct and indirect emissions. Indirect emissions occur from the volatilization of ammonia and nitrogen (di)oxide and the leaching of nitrate. To calculate N_2_O emissions, the fertilizer or organic amendment (kg) applied is multiplied by the respective emission factor, which varied depending on the type of N fertilizer or organic amendment applied and the climate zone. Emissions from the production of artificial fertilizer were included on the basis of the eco-invent database (Eco-invent). N_2_O emissions from drained inland organic soils was further included in the emission calculation on the basis of the type of land use, climate zone and soil type (peat/no peat)^45,50,65^. CH_4_ emissions from rice were excluded as rice cultivation was neglectable. CO_2_ emissions from soils and cropping were not considered.

#### GHG emissions from compost

Composting food waste results in N_2_O and CH_4_ emissions. N_2_O emissions were calculated on the basis of the N content of the food waste and then converted to N_2_O. CH_4_ emissions were based on the initial N content and then converted to carbon based on the C:N ratio of an average compost of 15. Finally, the carbon content was converted to total amount of emitted CH_4_ (ref. ^66^).

#### GHG emissions from transportation

Transporting crops, fish, food, by-products, manure and food waste results in CO_2_ emissions from the burning of fossil fuels. Distances from country to country (centre point to centre point) were quantified and the number of ton-kilometres quantified. Total ton-kilometres were multiplied by an emission factor (Eco-invent).

GHG emissions were summed into carbon dioxide equivalents (CO_2_e; 100 year time horizon, 28 for biogenic CH_4_ and 265 for N_2_O) to calculate total GHG emissions. Results were given in GHG emission totals for the EU and per capita per year. For the AgriBase scenario, the crop areas were calibrated to precisely match the current area per crop and land use type on a country level, therefore emissions of the AgriBase represent the current situation and are in line with FAOSTAT. Note, in all scenarios emissions are calculated and not optimized, but a constraint of 1,500 kg of CO_2_e per person per year per country was implemented to avoid random transportation.

## Supplementary information

Supplementary Information Additional figures presenting the results at different aggregation levels.

Supplementary Data Raw data underlying the figures within the paper.

## Data Availability

The raw data have been deposited in the GIT repository and are available on request under a licence similar to Creative Commons Attribution-Non Commercial-Share A like 4.0 International Public License. A dashboard is available on www.circularfoodsystems.org providing detailed data related to the results of this publication.

## Code availability

The model code has been deposited in the GIT repository and is available on request under a licence similar to Creative Commons Attribution-Non Commercial-Share A like 4.0 International Public License.
