# Supplementary material for: Circularity in Europe strengthens the sustainability of the global food system
Source: Nat Food. 2023 Apr 17;4(4):320–30. doi: 10.1038/s43016-023-00734-9 (PMC10154194; doi:10.1038/s43016-023-00734-9)
Supplement: Supplementary file 1 — Additional figures presenting the results at different aggregation levels. [file 43016_2023_734_MOESM1_ESM.pdf]

nature food

Article https://doi.org/10.1038/s43016-023-00734-9

Circularity in Europe strengthens the sustainability of the global food system

In the format provided by the authors and unedited

Supplementary Figures – Van Zanten et al. 2023

This document contains additional figures. More detailed information is available on www.circularfoodsystems.org including additional model runs to test the sensitivity of the model.

Table of Contents

Supplementary figures 2
- Crops 2
  - Agricultural land area 2
  - Crop yield 4
- Fertilization 4
  - Fertilizer sources 4
- Animals 6
  - Relative change in animal yield 6
  - Feed consumption and composition 7
- Humans 9
- Food consumption 9
  - Protein Consumption 11
  - Nutrient consumption 13
- GHG emissions 14
- Transport 14

Supplementary figures

This section contains supplementary figures related to the scenarios described in the article.

Crops

This section contains model results concerning crop area and crop yield.

Agricultural land area

Agricultural land area (sum of crop and grass land) is shown on an aggregated EU28 level. There are separate figures on an aggregated crop- and grass-land level and on aggregated crop-group level.

Total

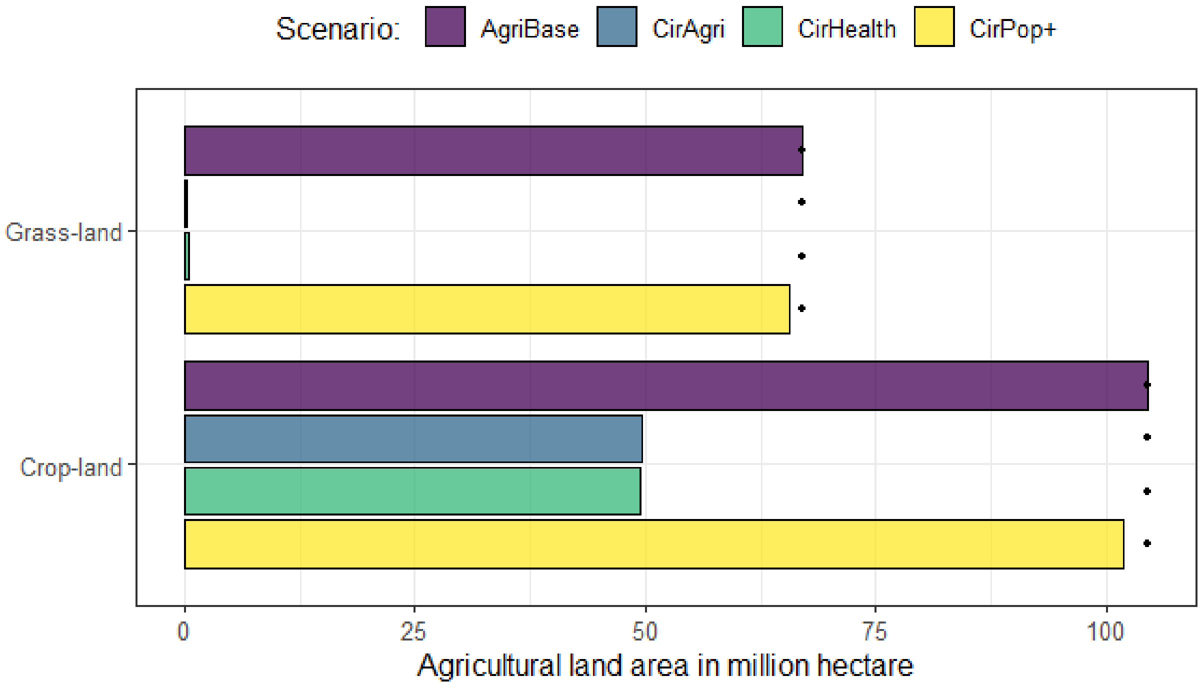

Supplemental Figure 1: Agricultural land area in million ha. Dots indicate the current agricultural land areas based on FAOstat data.

Crop group level

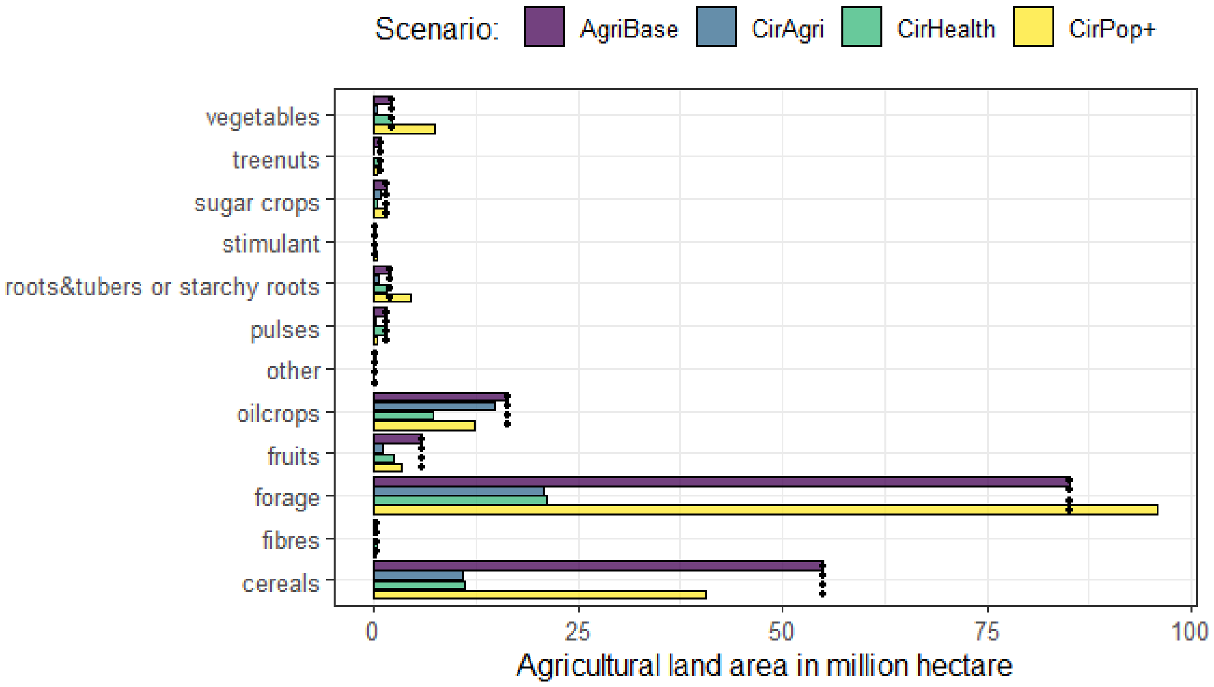

Supplemental Figure 2: Agricultural land area per crop-group in million ha. Dots indicate the current agricultural land areas based on FAOstat data.

Crop yield

Crop yield, including gras yield, is shown on an aggregated EU28 level.

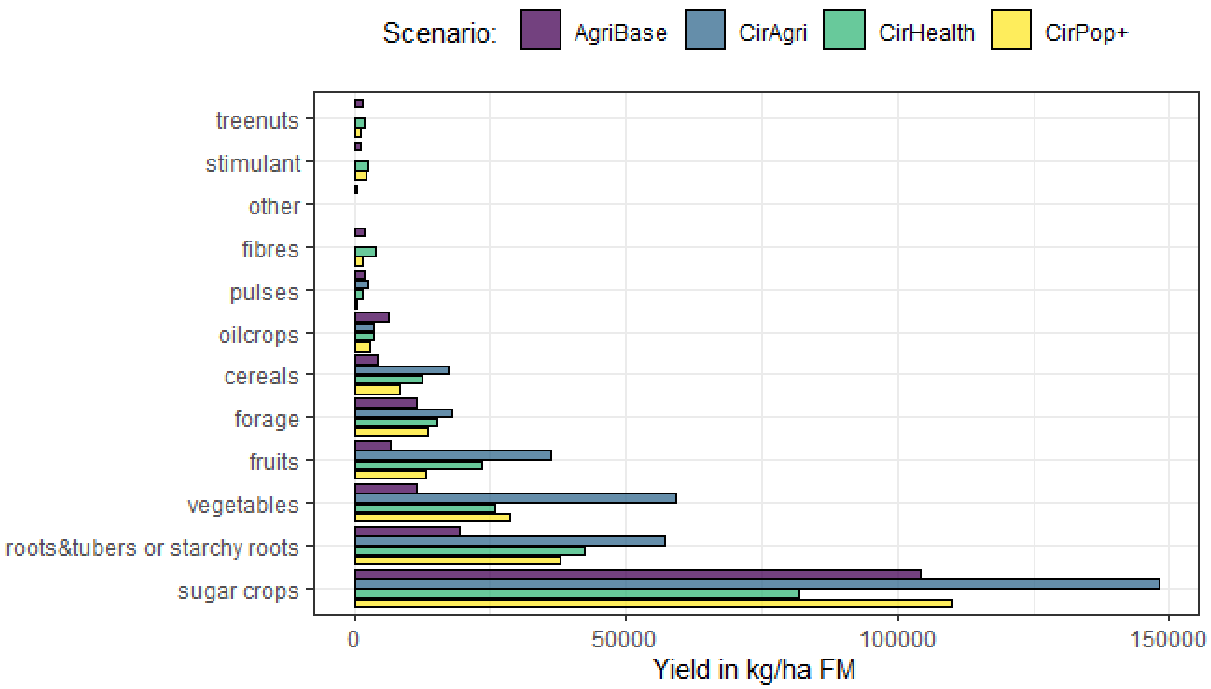

Supplemental Figure 3: Crop yield per crop-group in kg fresh matter per ha.

Fertilization

This section contains model results concerning crop fertilization

Fertilizer sources

This subsection contains figures indicating the sources of fertilizer, as well as the receiving crop groups and crops.

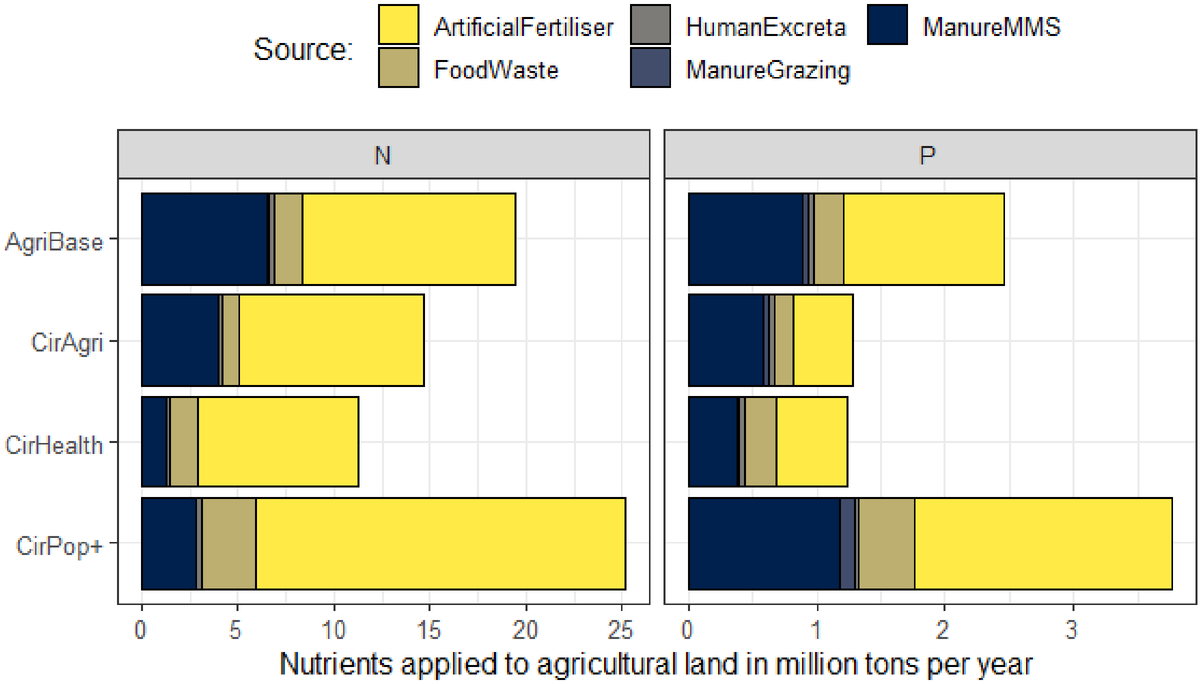

Supplemental Figure 4: Total fertilizer nutrients applied in million tons per year.

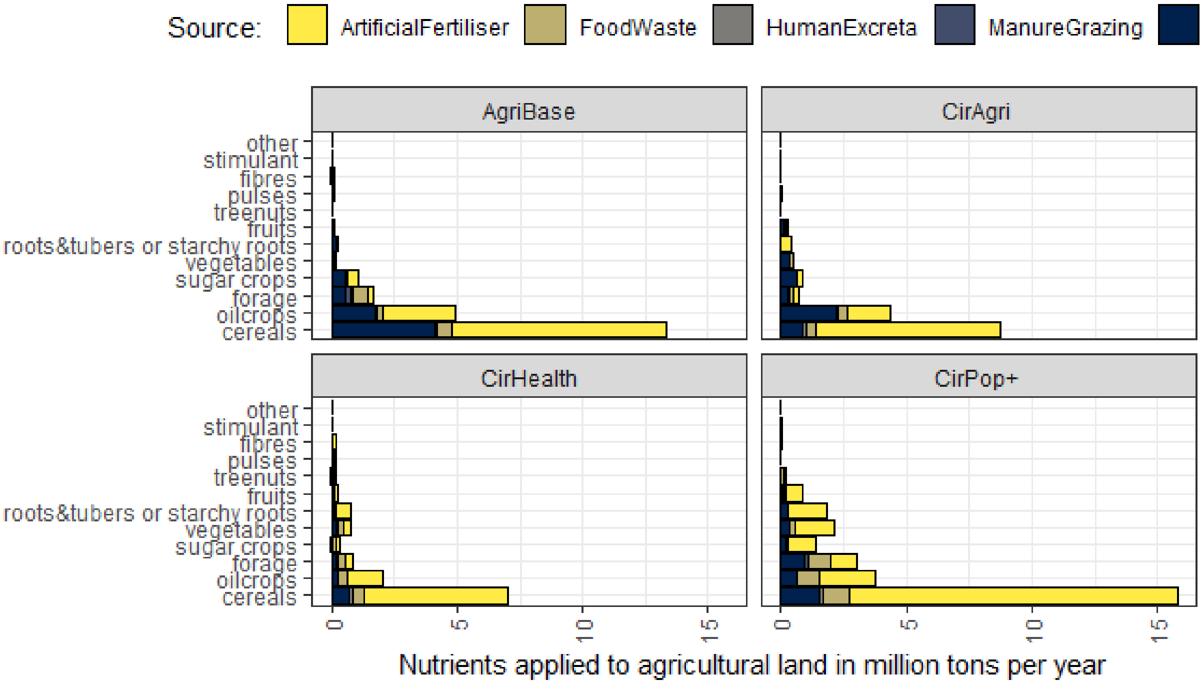

Supplemental Figure 5: Fertilizer nutrients applied per crop-group in million tons per year.

Animals

Model results concerning the animal subsystem are given in a figure with the change of animal yield relative to the baseline scenario. This figure is given on an aggregated EU28 level. A second figure shows the consumption and composition of feed on an aggregated EU28 level.

Relative change in animal yield

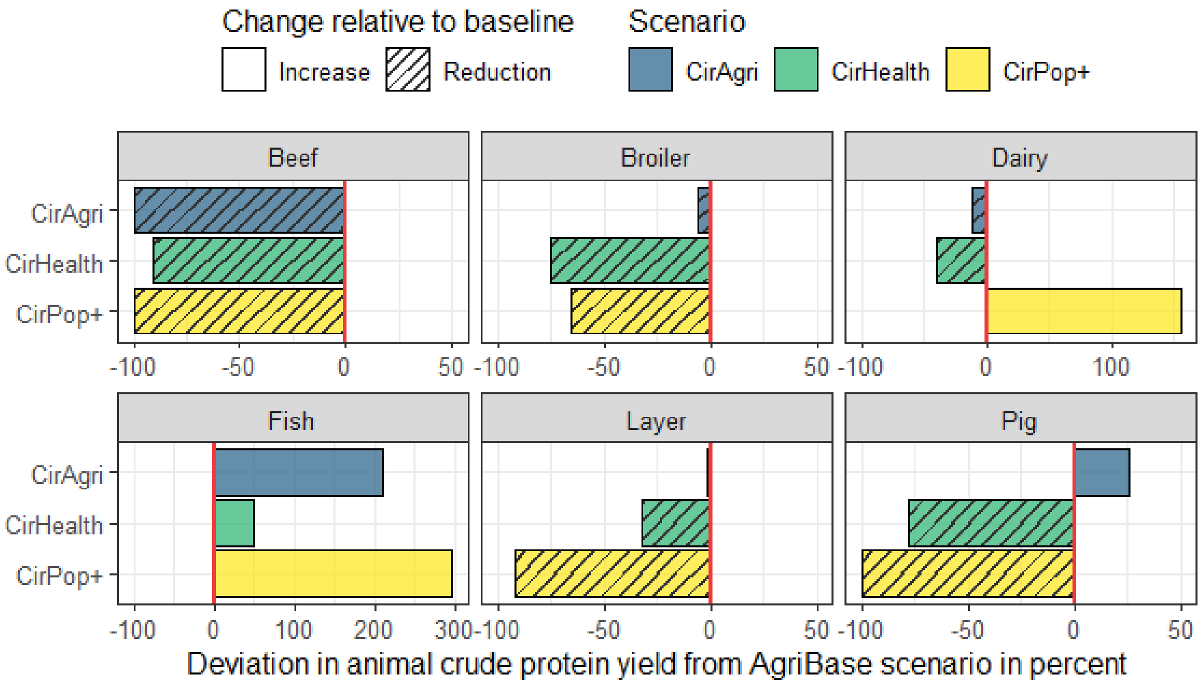

Supplemental Figure 6: Animal yield in crude protein. The change in animal yield relative to the baseline scenario, indicated by the vertical line, in percent.

Feed consumption and composition

Feed consumption and composition figures indicate the amount and types of consumed feed. There are separate figures for the total feed consumption of all animal production systems, the total feed consumption per animal production system, and the feed consumption of producers per animal production system relative to the number of producers.

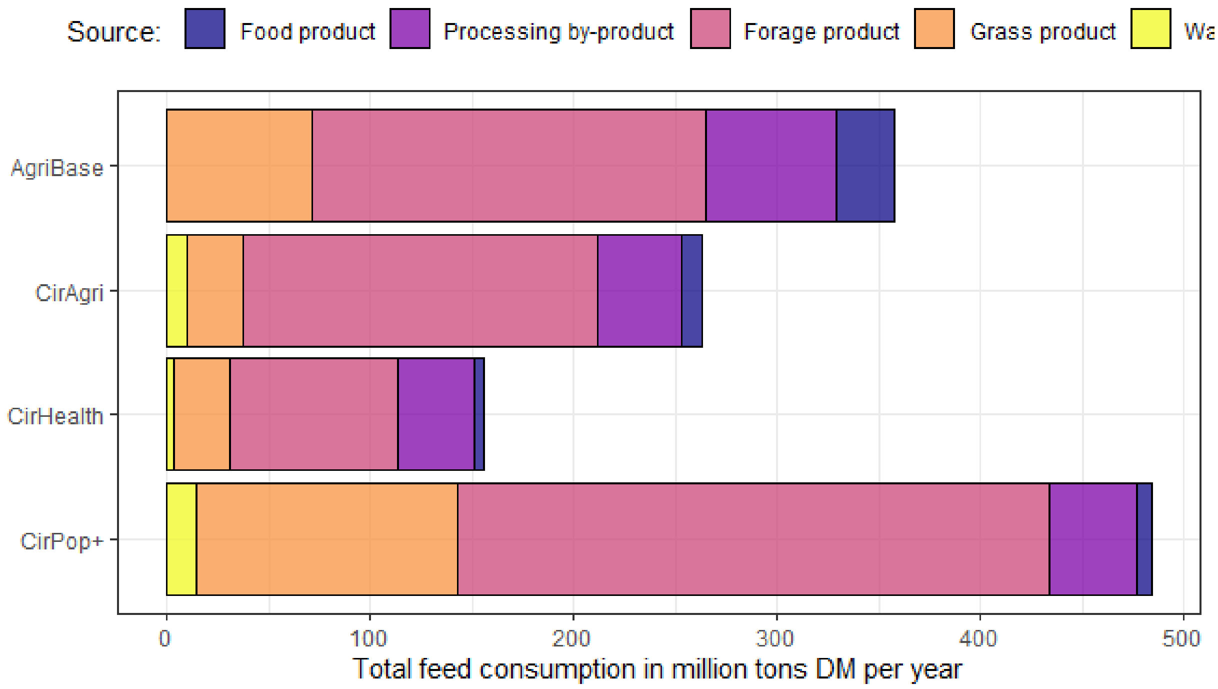

Supplemental Figure 7: Total feed consumption in million tons dry matter per year.

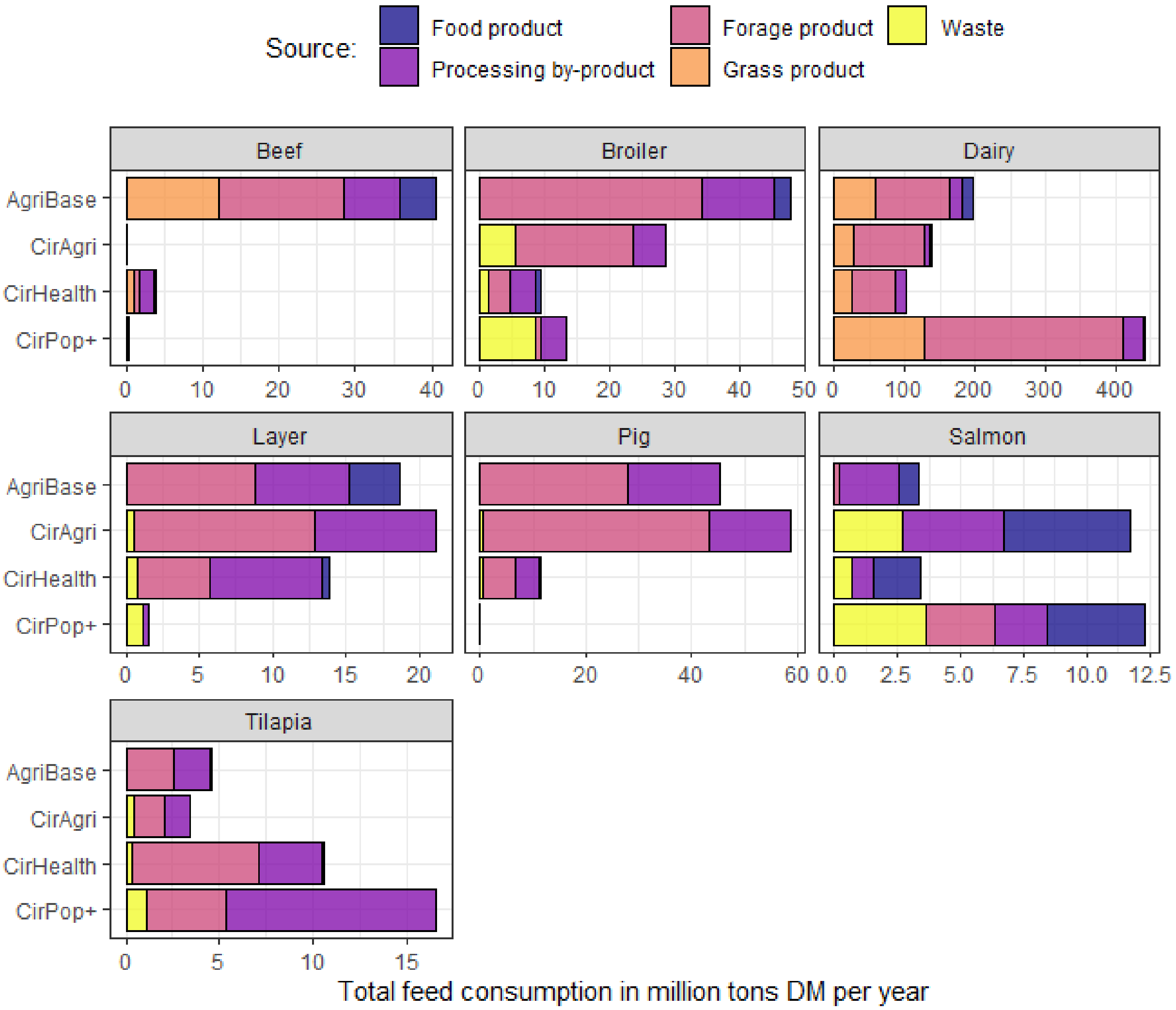

Supplemental Figure 8: Total feed consumption per animal production system in million tons dry matter per year.

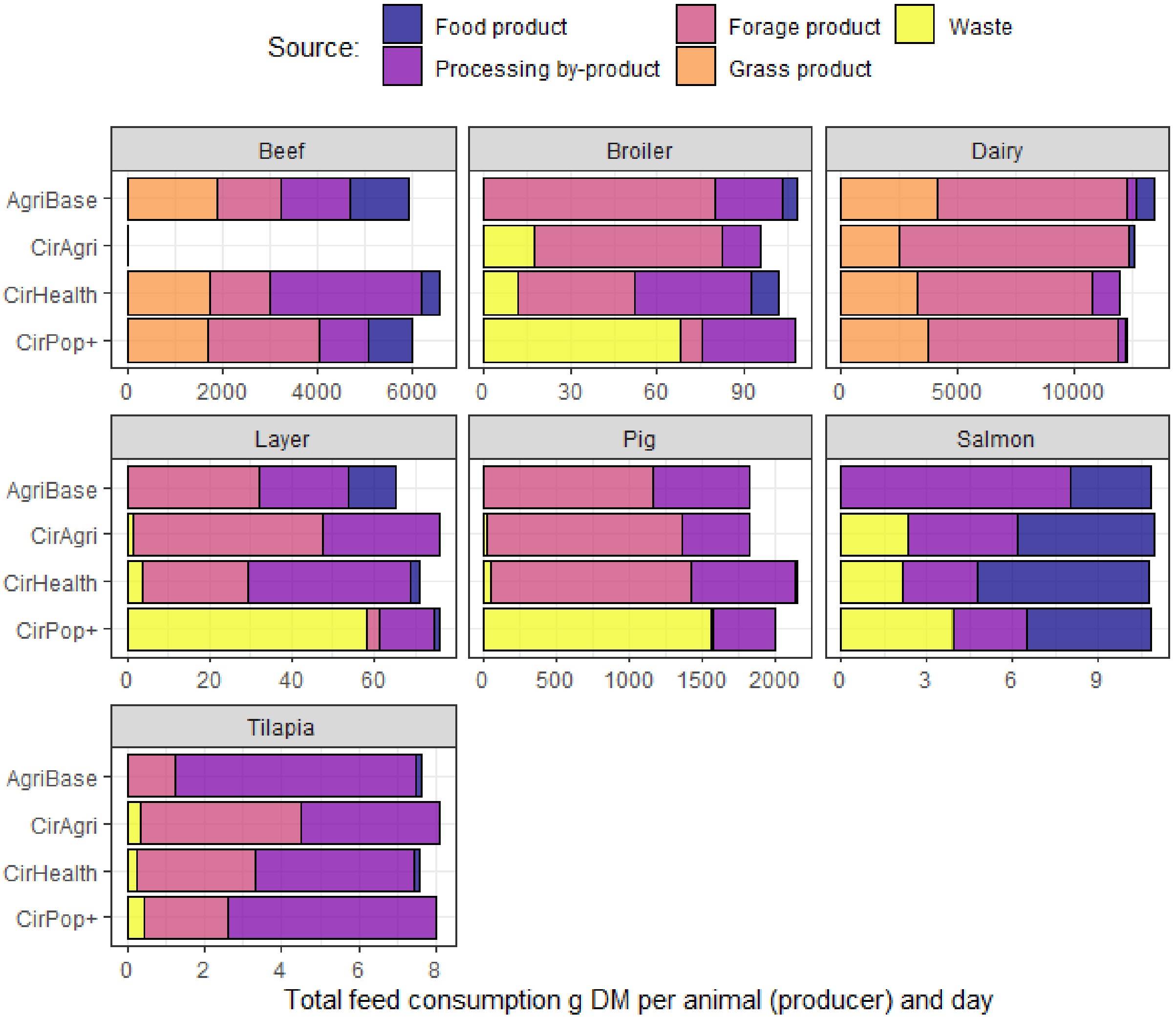

Supplemental Figure 9: Total feed consumption of producers per animal production system in g dry matter per animal (producer) per day.

Humans

This section contains model results concerning human consumption per food-groups and nutrients.

Food consumption

Food consumption figures are given on an aggregated EU28 level. There are separate figures for the total food consumption and the food-group consumption.

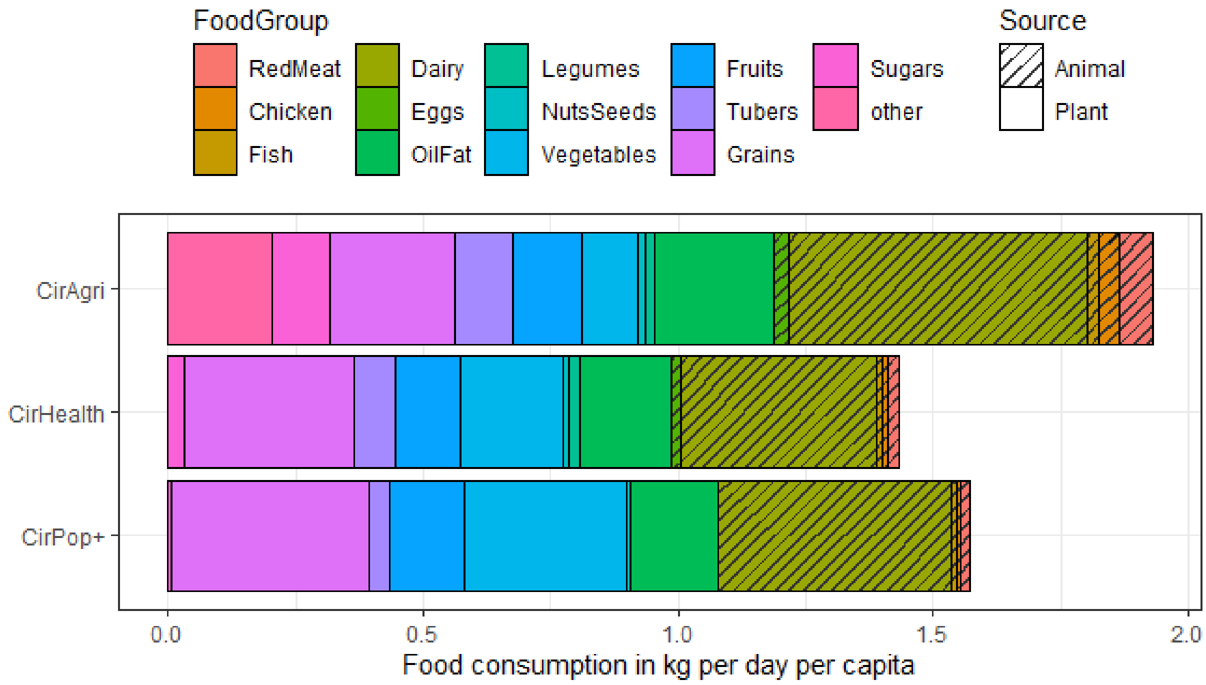

Supplemental Figure 10: Total food consumption in kg fresh matter per day per capita per scenario. The pattern differentiates between animal and plant sourced proteins.

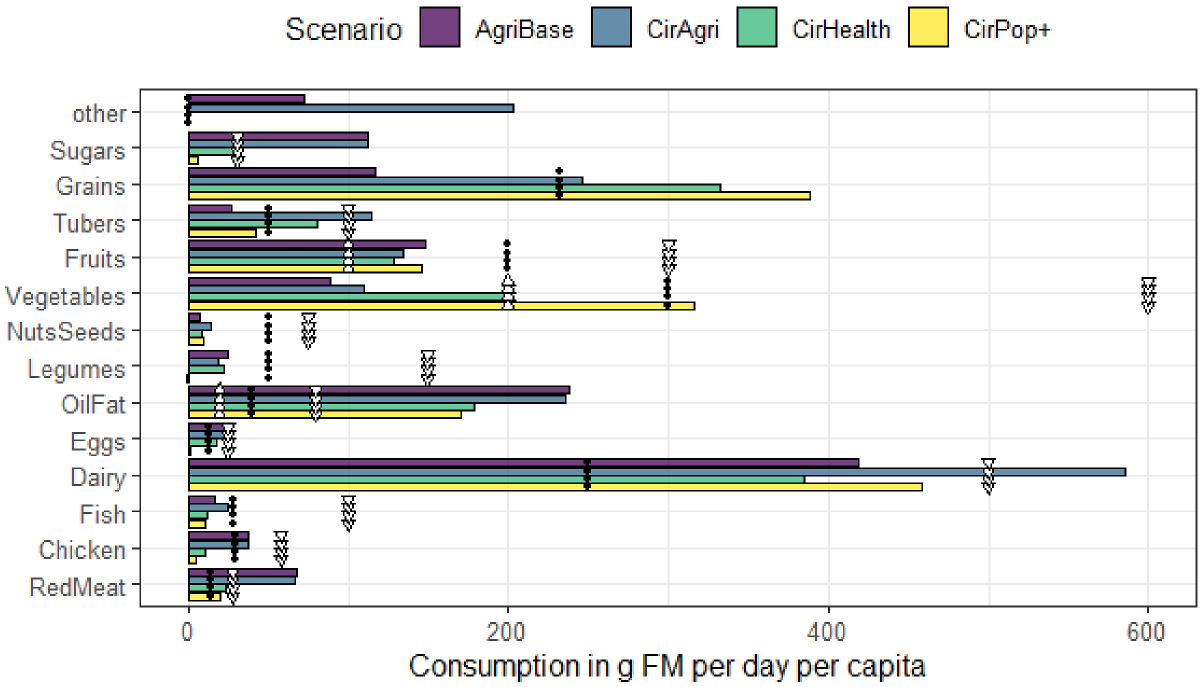

Supplemental Figure 11: Food-group consumption in g fresh matter per day per capita. The dots indicate the reference diet recommendations by the EAT-Lancet. The triangles indicate the minimum (triangle pointing up) and maximum (triangle pointing down) diet recommendations by the EAT-Lancet.

Protein Consumption

Food protein consumption figures are given on an aggregated EU28 level. There are separate figures for the total protein consumption and the protein consumption per food-group.

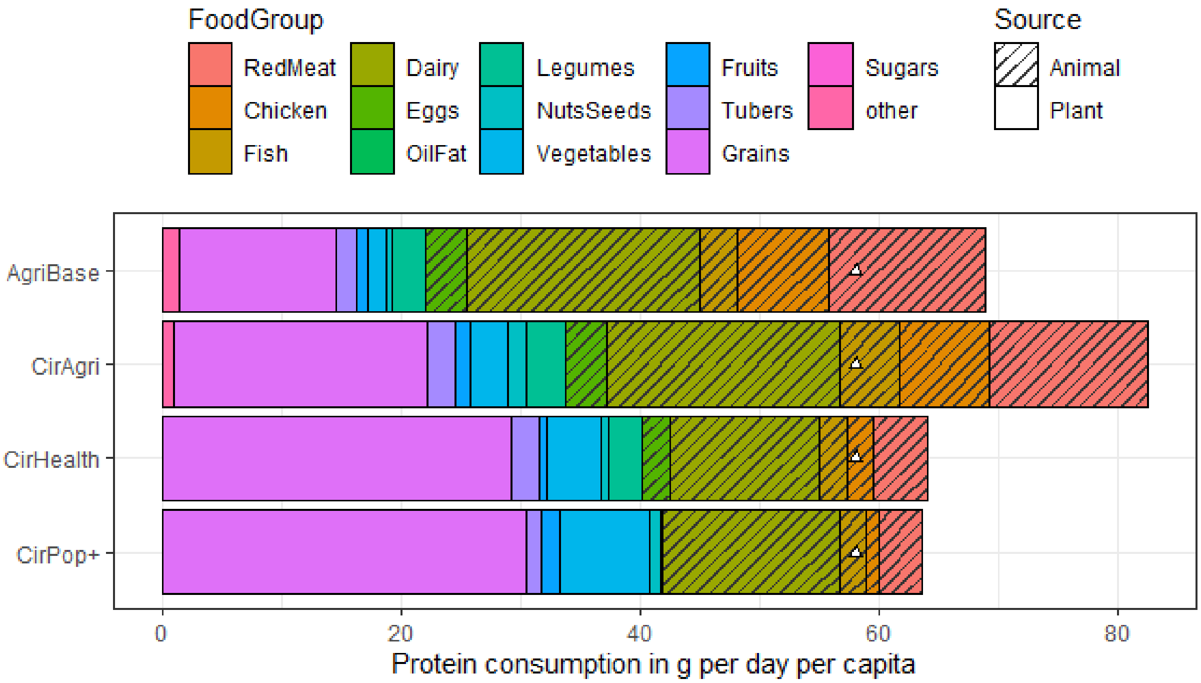

Supplemental Figure 12: Total protein consumption in g per day per capita. The triangles indicate the minimum (triangle pointing up) and maximum (triangle pointing down) protein consumption recommendations. The pattern differentiates between animal and plant sourced proteins.

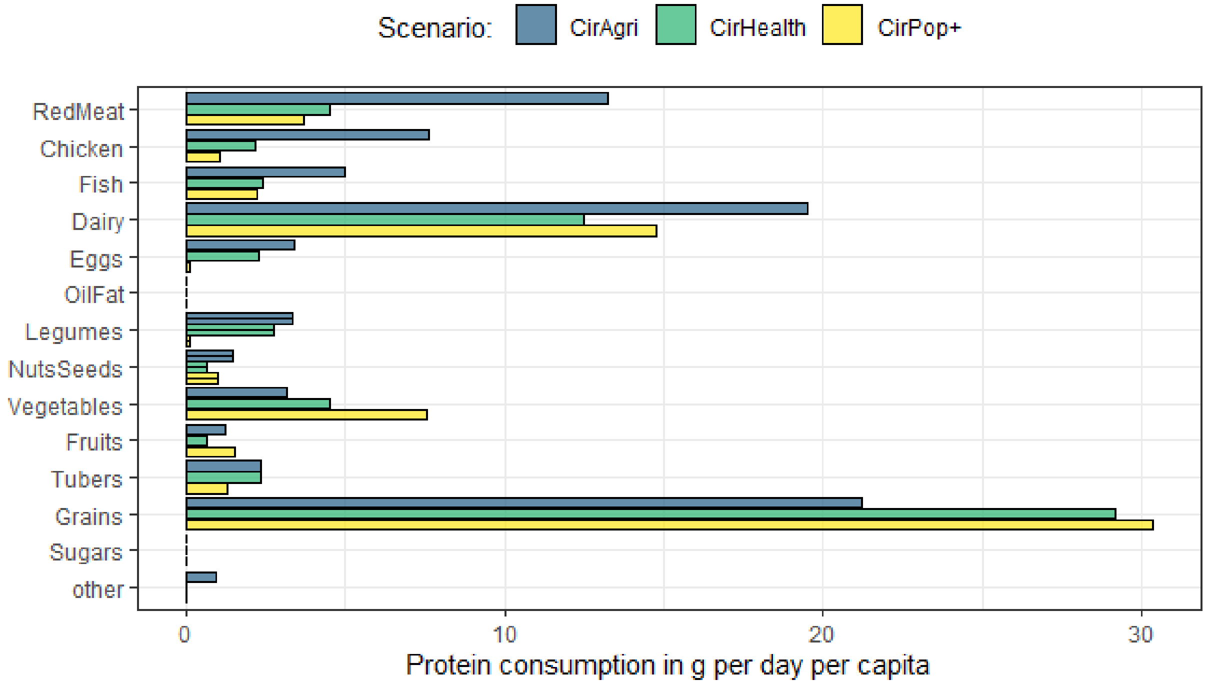

Supplemental Figure 13: Food-group protein consumption in g per day per capita.

Nutrient consumption

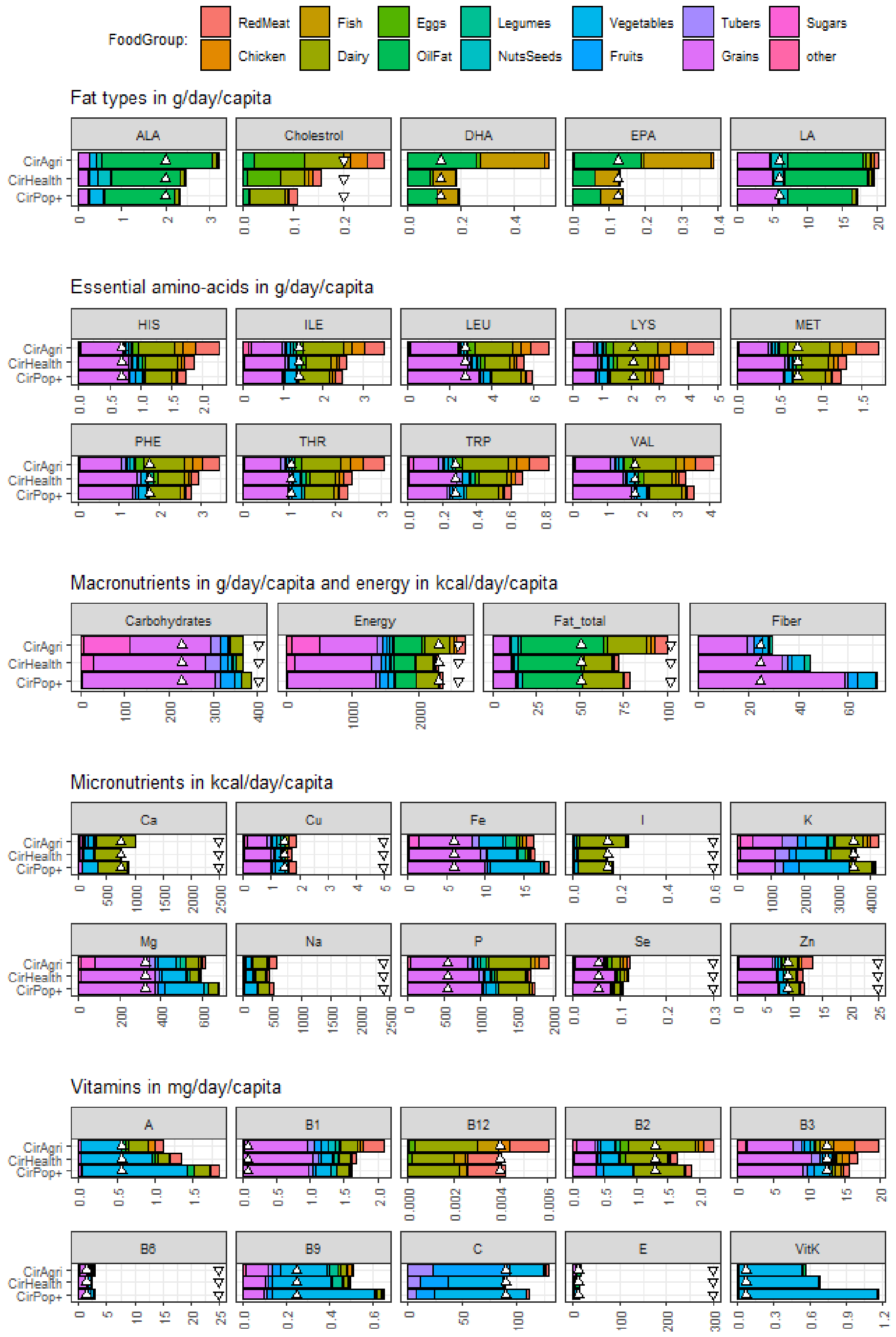

Supplemental Figure 14: Nutrient consumption in nutrient dependent weight units per day per capita, energy consumption is given in kcal per day per capita. The triangles indicate the minimum (triangle pointing up) and maximum (triangle pointing down) nutritional requirements, where applicable.

GHG emissions

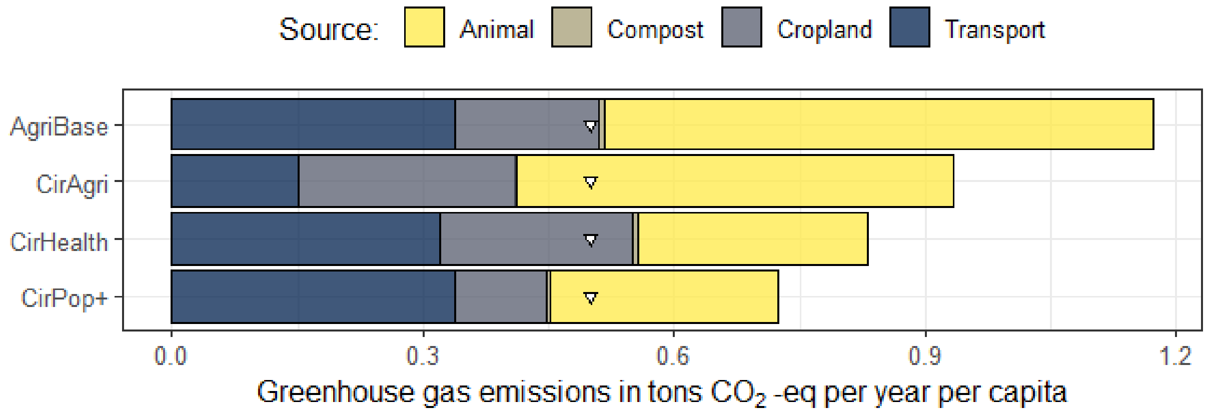

Supplemental Figure 15: The food system's greenhouse gas emissions in ton CO_2-equivalents per year per capita on an aggregated EU28 level. Triangles indicate the food system's maximum greenhouse gas emissions recommended by the EAT-Lancet, 500 kg CO_2-equivalents per year per capita.

Transport

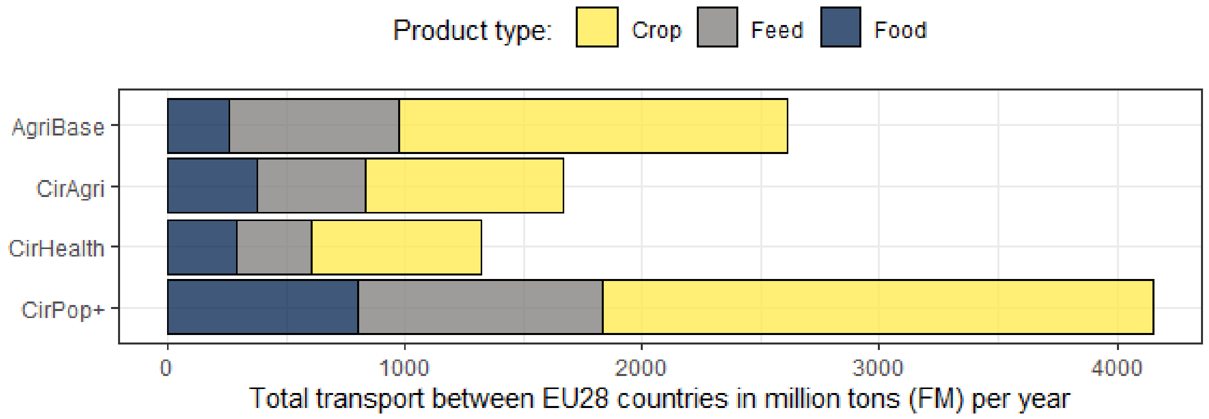

Supplemental Figure 16: Transport of food, feed, and crop products within Europe in million tons fresh matter per year.
